# In-service training and teaching resource proficiency amongst Chemistry teachers: the mediating role of teacher collaboration

**DOI:** 10.1016/j.heliyon.2021.e06995

**Published:** 2021-05-08

**Authors:** Jimmi Copriady, Hutkemri Zulnaidi, Masnaini Alimin, Sri Wilda Albeta

**Affiliations:** a Department of Chemistry Education Program, Faculty of Education, Riau University, Pekanbaru, Indonesia; b Department of Mathematics and Science Education, Faculty of Education, University Malaya, 50603, Kuala Lumpur, Malaysia

**Keywords:** Chemistry teachers, Teacher collaboration, Mediating role, Teaching resource proficiency, Training

## Abstract

This study aims to determine the level of collaboration, in-service training and teaching resource proficiency amongst Chemistry teachers by investigating the intermediary role of collaboration for training and teaching resources competency. A total of 184 Chemistry teachers in Riau, Indonesia, have participated in the survey. Using AMOS and SPSS 25.0 software to analyse the research data, we find a high level of collaboration, training and teaching resource proficiency amongst Chemistry teachers. Male teachers have higher proficiency level on all aspects than female teachers. The MANOVA test results show a significant difference in teacher resource competency based on gender. Male teachers are significantly more proficient than their female counterparts. However, they do not significantly differ in terms of collaboration and in-service training. The structural equation modelling test results show that collaboration has a significant role in Chemistry teachers’ involvement in training and teaching resources. These research findings encourage relevant parties to design effective collaborations amongst Chemistry teachers. They also offer new insights for Chemistry teachers to keep on mastering teaching resources nationally and internationally.

## 1 Introduction

The education system in Indonesia is always under close observations by many stakeholders and relevant parties. Although teacher reform is one of the government's educational goals, Allena et al. (2018) argued that it is an ambitious goal because the government only implemented the Teacher and Lecturer Law in 2005. Educational reformation is necessary because teaching and learning are processes that affect one's future. A solid foundation of education is a benchmark to measure and predict the progress of a country. Hence, teachers should have uplifting activities to enhance and expand their knowledge acquisition and skills. A competent teacher can influence his school quality and students' knowledge and experiences (Jussim and Harber, 2005). He should participate in professional communities via accessible communication networks to share information related to school subjects and education. He should have great attitudes and dedication in performing his duties and administering beneficial activities for students. Liu and Tsai (2017) argued that reforming education should be executed to encourage school teachers to be innovative in their teaching strategies and improving their teaching quality.

A teacher's education is a critical aspect to enhance his knowledge (Sexton, 2015). Ball, Thames and Phelps (2008) stated that knowing his lessons is a basic competence for a teacher to master. In other words, a teacher should master the curriculum contents comprehensively and focus on the most critical aspects. A superior Chemistry teacher can explain and provide detailed information by using various teaching aids. Murtaza et al. (2012) viewed a good teacher as one who is not only kind and committed but also honest with his profession and can fulfil his task enthusiastically. However, studies on teachers' subject mastery and mastery learning remain limited, especially in testing and identifying knowledge contents (Ball et al., 2008). A review by Allena et al. (2018) showed that Indonesian teaching environment has limited resources in local contexts. They added that this situation resulted in frustration amongst Indonesian teachers as they felt a sense of powerlessness in their local contexts. The training and laboratories provided for them failed to address these matters and remained inadequate. Young and novice teachers also reported the adverse influence of senior teachers in schools and a poor connection between curriculum design and pedagogical requirements expressed in an overloaded curriculum (Allena et al., 2018).

Vangrieken et al. (2015) regarded teacher collaboration as a critical aspect because effective collaboration encourages task interdependence, facilitating networking. Effective collaboration and discussions would enhance the socio-cognitive abilities of teachers (Liliane and Colete, 2009). Collaborative activities, such as between experienced and novice teachers, can be a platform for a roundtable discussion on personal representations, beliefs, theories, conceptions and teaching practices. Liu and Tsai (2017) added that collaborative experiences would provide a valuable reference for teachers. A collaborative design deals with matters of concern, as expressed in the saying: ‘It is never a process that begins from scratch, but to design is always to redesign’ (Voogt et al., 2015). Following this assumption, Fulton and Britton (2011) studied a group of Math and Science teachers by engaging them in discussions about their subjects. They found significant improvements amongst the teachers, who exhibited an understanding of their subjects, eagerness to teach and resourcefulness. However, no existing evidence supports the contribution of collaboration to teacher training or its efficiency as a teaching resource. Collaboration activities amongst teachers still suffer from the problem of uncoordinated processes. The discussion between teachers also tends to focus on superficial issues, including sharing of general experiences.

## 2 Research background

Chemistry teachers have difficulties and constraints in mastering their subject matters. They suffer from efficiency and productivity problems due to the quality of their academic services, particularly in terms of teaching methods, media and library services. Some chemistry teachers also experience academic restraints. For example, the interaction between academic staff and students, in general, only applies in the classroom (Abdullah et al., 2014; Liu and Tsai, 2017). Despite these challenges, teachers need to master and be proficient in their subjects. Hence, the administration should provide continuous and sustainable efforts to encourage Chemistry teachers to improve their proficiency level and develop their knowledge and Chemistry skills. Davis and Simmt (2006) argued that one could attain knowledge by using different concepts. Activities, such as establishing a communication network amongst Chemistry teachers as a community, can be a great avenue for teachers to share and compare information related to their subject matters. Those who can participate in a communication network can improve their visions and share their knowledge about Chemistry. Horn et al. (2017) stated that considerable collaboration amongst teachers has relation to their conversation concept and the role of division of responsibilities (Runhaar et al., 2014). The professional communities can continuously allow teachers to participate in educational forums, such as seminars and panel discussions.

Through a continuous series of training, teachers can improve their teaching quality. Fulton and Britton (2011) found that teachers who participated in workgroup conversation about the subject they teach understood better and felt more prepared to teach their subjects than those who did not participate. Their participation also helps them build an online academic community to enhance their teaching and learning abilities and the quality of their subjects. Teachers can improve their learning, knowledge, impression and faith when they participate in learning activities (Opfer and Pedder, 2011). They can also share their valuable experiences in mastering Chemistry teaching materials, especially about using the right methodology, such as experiments in the laboratory. Truijen et al. (2013) defined collaborative work as an ability of a team to work successfully or the capability of a community to work together.

As a complex task, the definition of teacher collaboration lacks consistency. Woodland et al. (2013) argued that teacher collaboration is inconsistent and often theoretical. Moreover, teacher community lacks a clear definition and is difficult to assess (Vangrieken et al., 2013). Teacher community is a group of socially interdependent teachers who participate in discussions, knowledge building and domain and goal sharing (Brouwer et al., 2012). Vangrieken et al. (2015) stated that teachers could share knowledge and furnish collegial support through collaboration. Teacher collaboration focuses on conversation and idea (Hargreaves & O'Connor, 2017). Mora-Ruano et al. (2018) provided another definition used exclusively at the teacher level. Teacher collaboration involves relational trust, school administration and coordination and the exchange of ideas and materials between teachers, which play a central role in having authority.

Teachers should help their students to engage all of their senses during the learning process (Muijs and Reynolds, 2010). Being conscious of the subject or lesson materials would help them understand and appreciate their lesson. Things keep changing every day, and therefore everybody needs to continue learning new things (Tovey and Lawlor, 2008). Even as professionals, teachers still have limitations and are inadequate in mastering the whole Chemistry knowledge. Mastering the subject matter is not easy, as one needs to improve his proficiency, escalate his knowledge and learn diverse skills continuously. Teachers should participate in enrichment activities through communication networks to develop good communication skills and attitudes. Copriady (2014) stated that teachers are generally great agents of educational development and innovations. They should continue to develop their knowledge and skills through networking and mutual sharing of academic resources.

A Chemistry teacher can only explain his teaching materials well if he masters his subject. A well-versed teacher can explain the lessons clearly to the students and be flexible with his teaching strategies. Hence, a teacher needs to master the subject that he is teaching. A teacher with low proficiency will dampen the quality of his teaching, which can only improve by mastering the teaching materials. Moreover, the subject that a teacher chooses to master is extremely important (Ültay, 2017). Suyono and Hariyanto (2011) stated that learning materials must serve the learning objectives and students' need. The learning materials should be relevant to the environment and ethical ideologies. They have to be well-organised and systematically logical and designed using a standardised book. The government and private sectors held several programmes, including in-service training on various aspects related to teaching resources proficiency and laboratory management, to enhance teachers’ professionalism. These training and programmes are conducted professionally by experts and lecturers. Yet, challenges and constraints still exist. Schools and higher learning institutions should establish a good relationship and cooperation to address these issues. This link and understanding can provide opportunities to form solid platforms for engagement in various training programmes and experiments (Suyanto, 2013).

Studies showed that teacher teamwork community has a positive effect on student outcome (Louis et al., 2010; Egodaeatte et al., 2011). De Jong et al. (2019) found that collaboration offers an opportunity for teacher learning depending on the context. Darling-Hammond et al. (2017) reported that teacher collaboration structure could improve student outcome through problem-solving and learning together. Thus, collaboration is a significant predictor to student outcomes (Reeves et al., 2017). Teachers can employ different collaborative activities to enhance the effectiveness of teaching and learning behaviour.

## 3 Research focus

Scholars investigated the advantages of teacher collaboration, such as professionalism development (Puchner and Taylor, 2006), improved learning strategies and cooperation between teachers and administrators, in understanding pedagogy to enhance teaching comprehension (Lau, 2013). Burke (2013) and Egodawatte et al. (2011) described several positive outcomes of professional collaboration, especially in the development of professional knowledge. Pawan and Ortloff (2011) concluded that teachers could produce more activities if they work collaboratively. However, an organised collaboration for teachers remains impossible with various challenges. The common hurdles are time constraint and personal beliefs in sharing their resources (Doppenberg, Bakx, & den Brok, 2012; Ning et al., 2015).

Pawan and Ortloff (2011) stated that inadequate procedures could provide less influence on teacher collaboration. Vangrieken et al. (2015) even showed some negative outturns of teacher collaboration. The noble initial ideas of collaboration backfired when it turned into a competition amongst teachers, and the programmes increased their workloads, thereby causing other conflicts.

Following the above discussion, further research on collaboration, training and teaching resource proficiency amongst Chemistry teachers is relevant. Scholars should further explore the influences of collaboration on efficient training, which can help teachers be proficient in providing teaching resources. They should also consider gender differences to obtain various perceptions of teachers’ needs based on gender. Thus, the current study aims to answer the following research questions:

1. What are the levels of collaboration, in-service training and teaching resource proficiency amongst Chemistry teachers?
2. Do teacher collaboration, training and proficiency have significant differences based on gender?
3. What are the effects of collaboration as a mediator in the relationship between training and teaching resource proficiency?

## 4 Methods

### 4.1 Research design

In this study, we surveyed Chemistry teachers in Riau, Indonesia. We designed a questionnaire to evaluate their perspectives on collaboration, in-service training and teaching resource proficiency. Following Creswell (2014), we employed a quantitative method because a ‘numeric description of trends, attitudes or opinions of a population can be obtained by studying a sample of that population’. A survey is a method to collect sample information by using a questionnaire that allows the respondents to answer independently (Chua, 2006). We used a questionnaire in this study to collect data on teacher collaboration, in-service training and teaching resource proficiency. A questionnaire is the most effective instrument for attitude studies when collecting data from a large group of respondents on a limited timeframe (Wiersma, 2000). A questionnaire allows people to express their responses freely. It also enables researchers to adjust the diversity of expressions and obtain consistent responses through standard questions. We considered this research design suitable for this study because it could easily generalise the results from the samples to the entire population (Chua, 2016).

### 4.2 Participants

The population was essentially the target from which the researchers selected the sample from; it comprises the totality of individuals (elements) about which the research is concerned (Ary et al., 2014). The sample was essentially the population targeted; researchers limit their study sample to a manageable size considering various parameters, such as the statistical technique used in data analysis, framework/model of the study, time, and financial constraint. In this study, 184 Chemistry teachers in Riau, Indonesia, participated in the survey. All respondents had attended the subject teacher forum (MGMP) in 2019, which is organized every month for all teachers in every district in Riau Province, Indonesia. The forum discussed the foundation of collaborative learning for teachers and knowledge mastery in the field of chemistry. The questionnaires were distributed to all attendees to respond for 30 min upon completing the forum. The distributed questionnaires received a return of 184 as several questionnaires were dropped due to incomplete data. Hence, only completed questionnaires were analyzed to answer the research questions of this study. Amongst them, 50 teachers were males, and the remaining 134 were female. We randomly selected the participants using the sample size table proposed by Krejcie and Morgan (1970). Patton (1990) argued that no rules exist in determining sample size. However, the large sample size is preferable when research uses a survey questionnaire as the research instrument. Sudman (1976) claimed that each major group or subgroup in the sample should have a minimum of 100 participants. This study received approval from the Pekanbaru City Education Office and the University of Riau. We provided the survey questionnaire to the school principals and the lecturers at the university. They helped distribute the questionnaire amongst chemistry teachers who have to continue postgraduate programmes to the University in Riau, Indonesia. For ethical purposes, we also included a note in the questionnaire that the answers from the study sample will remain confidential and be used only for research purposes.

### 4.3 Research instrument and procedures

We adapted the items for measuring collaboration from Copriady et al. (2018). We used a five-item scale (i.e 1. Strongly Disagree; 2. Disagree; 3. Neutral; 4. Agree and 5. Strongly Agree). These items were about collaborating with other teachers from other schools and communicating with colleagues to solve problems related to students. We designed 30 items to measure collaboration (See Table 1). We also adapted the elements for measuring in-service training and proficiency from Copriady et al. (2018). We used a five-item scale (i.e 1. Strongly Disagree; 2. Disagree; 3. Neutral; 4. Agree and 5. Strongly Agree). We designed 35 items to measure in-service training focusing on training organised by the school and the ministry, which improves understanding and literature review on academic writings in accordance with the Information Communication Technology (ICT) (See Table 2). We designed 32 items to measure proficiency focusing on teaching aids and materials, lesson materials and subject matter (See Table 3). We used a three-item scale (i.e 1. Disagree; 2. Neutral; 3. Agree). Before assigning the instruments to the samples, the researchers constructed the instruments used to evaluate these variables and consulted with experts to verify the instruments. According to Gay and Airasian (2003), conduct a questionnaire validity test to strengthen language use and improve clarity and project content. All three experts who participated in this study agreed that the instrument is suitable for measuring variables. We conducted a pilot study amongst 60 Chemistry teachers before the actual study. We intentionally selected initial respondents who had similar characteristics to the actual respondents to measure the reliability of the instrument. In this study, a Cronbach's alpha value in between 0.70 and 0.73. Hair et al. (2010) stipulated the value of 0.70 is acceptable. This high-reliability value implied that each item was feasible for the actual study.

**Table 1:** Teacher collaboration.

No	Item
1	I am committed to make interschools collaboration successful
2	I am good at ICT to carry out collaborative networks with other schools
3	I am not competent in using ICT to establish relationships with others.
4	I have expertise in improving student achievement after joining collaborative networks.
5	I lack the commitment to implement collaborative network with other schools
6	I am very involved in activities or programs organized by the school
7	I will cooperate and involve truthfully in collaborative programs with other schools.
8	I have good relationships and interactions with others
9	Expert teachers have to share their thoughts and knowledge on various interschool excellent programs
10	I do not want to share ideas and knowledge with other teachers.
11	Collaborative culture is less applied in organizations
12	Educational collaboration is only available in certain schools
13	Information websites are unavailable to provide guidelines for interschool collaborative programs
14	I share ideas on the web through the use of ICT as a way to improve student achievement.
15	I often provide detail explanation on work instructions to colleagues
16	I like to share my opinions when I am in a meeting
17	I have the opportunity to enhance my knowledge in excellent programs through collaboration with other schools.
18	I have the opportunity to improve my work performance in excellent programs through collaboration with other schools
19	A module or a manual on how to conduct collaborative programs should be made available.
20	Group discussions can help me improve the quality of learning
21	I often ask other experienced teachers whenever I have problems with students in the learning process
22	I often have communication problems with other teachers
23	I like to share my learning problems with other teachers
24	I prefer to solve my learning problems on my own
25	I like to solve my learning problems with my colleagues
26	I like to discuss with several teachers to solve any learning problems
27	I feel that meetings with other chemistry teachers are not very interesting.
28	Appropriate facilities and conducive infrastructure can assist teachers in implementing collaborative programs
29	Interschool Collaborative programs are expensive
30	Schools provide ICT to facilitate the success of collaborative networks

**Table 2:** In-service training.

No	Item
1	Training materials are useful for me as a teacher to implement learning improvements
2	Training materials support me in carrying out my duties
3	Training aids and materials can be used to enhance knowledge and can be applied in my school
4	The learning method used by the tutor during training can be applied at my workplace
5	The trainings that I have attended can improve my students' understanding of the material I delivered during learning activities
6	The training that I have received can improve my students' learning outcomes
7	The training that I have received can enhance my students' motivation to participate in learning activities at school
8	The training I have received can increase students active participation in learning activities at school
9	The outcomes of my training are innovations in learning activities at my school.
10	The trainings that I have attended can contribute ideas to improve the quality of education in my school
11	Curriculum training can accommodate the targeted aspects of the training
12	Curriculum training can develop a comfortable and conducive learning atmosphere for participants
13	My self-confidence has improved after completed my curriculum training.
14	Training is an effort to improve teachers' skills
15	I am active in participating in various trainings to improve my understanding on teaching.
16	Job specific skills are improved due to regular training.
17	The ability to cooperate with other teachers improved after attended a training program
18	Trainings on academic writing helped me to generate more ideas to carry out an action research.
19	Trainings on academic writing helped me to compile instructional materials/learning module.
20	I can optimize the use of ICT better, after participated in ICT training
21	ICT-based training using chemical software (Chemsketch, isisdraw, etc.) helps me to design teaching materials.
22	ICT-based training provides new knowledge to communicate with other chemistry teachers.
23	ICT-based training enriches me with knowledge on how to access educational materials online.
24	ICT-based training helps me to get the latest knowledge on teaching materials at school.
25	ICT based training helps me to get more ideas to conduct classroom action research.
26	ICT based training helps me to acquire the latest teaching methods and strategies.
27	Laboratory training helps me to plan appropriate practicum guides according to the curriculum.
28	Laboratory training helps me to design practical work procedures for each material.
29	Laboratory training helps me to create work safety procedures in the laboratory
30	Laboratory training helps me to create task sheets/handbook based on the textbooks
31	Laboratory training helps me to determine the suitable tools and materials to be used in a practicum.
32	Laboratory training is only for teachers who teach practicums.
33	Laboratory training makes it easier for me to carry out a practicum.
34	Laboratory training makes it easier for me to arrange practicum tools.
35	Laboratory training makes it easier for me to understand the working principles of laboratory equipment.

**Table 3:** Proficiency.

No	Item
1	I can relate subject matters with the current development
2	I convey the subject matter well
3	I always prepare the teaching aids and materials prior to the lesson
4	I prepare continual teaching materials.
5	I use appropriate lesson materials based on the learning objectives.
6	Lesson materials should be relevant with the development of knowledge
7	The lesson materials should include factual matters
8	The lesson materials should include conceptual matters
9	The fruit juice industry adds polyphenols to avoid oxidation
10	When sliced, red onions will release volatile compounds
11	The presence of CO in the human body reduces the ability of the blood to bind oxygen
12	Buffer solution is used to maintain pH at a certain value in various chemical applications, one of which is the bicarbonate buffer system which regulates PH in the blood.
13	Nylon is an example of the end result of condensation polymerization
14	Enzymes are catalysts.
15	Proust's law states that the mass element that makes up a compound is always constant in composition.
16	The saponification reaction is an oil/fat hydrolysis reaction using strong acids.
17	The catalyst serves to increase the activation energy
18	Ketones can be oxidized to carboxylic acids
19	The atoms of the metal element release their valence electrons to form ion^+x^
20	Polyatomic symmetry molecules that have a central atom without lone pair electrons are always polar.
21	All strong electrolyte solutions are ionizable and have an ionization degree of 0 <α < 1
22	In an exothermic reaction, the products have a more stable energy than the reactants, because the energy of the products is lower than the reactants.
23	The anode of the Galvanic cell is positively charged because the anode attracts the anion from the solution while the cathode is negative.
24	In an absorption process, a substance (material or energy) is distributed to other substances. While in adsorption, the interaction occurs only at the surface level of the same substance.
25	C5H12 is definitely a Pentane compound
26	I have to teach students the atomic structure first before introducing the hydrocarbon compound material
27	I have to teach students the structure of atoms and hydrocarbon compounds prior to chemical bonding material.
28	Students must master stoichiometric material before I teach solutions.
29	Atomic structure material is a prerequisite for mastering stoichiometry
30	Students must master stoichiometric material before I teach solution equilibrium.
31	I have to teach stoichiometry before teaching reaction rate material
32	Stoichiometry material is a prerequisite for mastering electrochemistry

### 4.4 Data analysis

We used SPSS 25 and AMOS 23 software for data analysis. We conducted descriptive statistics using SPSS software to determine the levels of collaboration, training and teaching resource proficiency amongst Chemistry teachers. We also carried out a MANOVA test to identify any contrasting condition on teacher training, collaboration and teaching resource proficiency amongst the respondents based on gender. MANOVA's analysis results can determine the homogeneity or differences of variances when a research situation involves many dependent variables (Pallant, 2007). We used structural equation modelling (SEM) to associate the function of collaboration in assisting training and teaching resource proficiency amongst Chemistry teachers. We referred to Dearing and Hamilton (2006) for the intermediary analysis in this study. We also conducted chi-square test to assess the null hypotheses with some other measurements, including comparative fit index (CFI), the goodness-of-fit index (GFI) and root mean square error of approximation (RMSEA). We conducted these tests to determine the reliability value of the research model. Researchers should conduct at least one type of compatibility index tests from the category of absolute fit index (e.g. Chisq, GFI and RMSEA), incremental fit index (e.g. AGFI, CFI and TLI) and parsimonious fit (Chisq/df; Awang, 2012). We performed a bootstrapping procedure by implementing a bias-corrected percentile method (Guan, 2003) to analyse indirect effects. A maximum likelihood bootstrapping procedure applies for research with a bootstrap sample of 1000 and a bias correction confidence interval of 95% (Mohamad et al., 2018). The bootstrapping procedure in the AMOS programme is fit to manage non-normal data (Byrne, 2013).

## 5 Results

### 5.1 Levels of teacher collaboration, teacher training and teaching resource proficiency

Table 4 shows the mean value and standard deviation to explain the connection between the variables in this study. The descriptive analysis results showed that the level of in-service training was high (mean = 3.98), and the level for teacher collaboration and teaching resource proficiency was moderate (mean = 3.51 and 2.49, respectively). The Pearson correlation of the studied variables showed a positive correlation in the range of 0.38–0.52 with a moderate strength of relationships.

**Table 4:** Mean, standard deviation and the relationship amongst variables.

Variable		1	2	3
1	Teacher collaboration	1		
2	Training	0.52	1	
3	Proficiency	0.37	0.41	1
Mean		3.51	3.98	2.49
Standard deviation		0.21	0.35	0.20
Internal consistency		0.70	0.93	0.71

### 5.2 Differences in teacher collaboration, in-service training and teaching resource proficiency based on gender

We conducted a MANOVA test to differentiate the state of teacher collaboration, in-service training and teaching resource proficiency between the male and female Chemistry teachers. This study satisfied all the required conditions for carrying out the MANOVA test before the actual test. The Box’M test results showed no significant variant–covariant amongst the dependent variables for all independent variable levels, with *F* values = 2.22 and sig = 0.039 (*p* > 0.001). Thus, the variant–covariant dependent variables were homogeneous across the independent variables. On the basis of the above conditions, we conducted a MANOVA test to determine the differences in teacher collaboration, training and resource proficiency. The Wilk's Lambda test results revealed the value = 0.97, *F* (1, 182) = 2.07 and sig = 0.106 (*p* > 0.05). The eta-squared test results showed a value of 0.03, implying a small or minor effect of differences (Cohen, 1988). Overall, the test results showed no significant difference between the male and female teachers in terms of teacher collaboration, training and teaching resource competencies.

Table 5 shows no significant differences in teacher collaboration (*F* = 0.68 and sig = 0.41, *p* > 0.05) and in-service training (*F* = 0.68 and sig = 0.41, *p* > 0.05) based on gender. The eta-squared values for teacher collaboration (0.01) and in-service training (0.01) showed a small effect of differences (Cohen, 1988). By contrast, a significant difference was evident in teaching resource proficiency (*F* = 6.23 and sig = 0.01, *p* < 0.05) based on gender. The eta-squared value of gender difference in teaching resource proficiency was also small (0.03). In terms of mean values, male teachers (mean = 2.54) had a higher proficiency level than female teachers (mean = 2.46).

**Table 5:** MANOVA on the differences in teacher collaboration, in-service training and teaching resource proficiency based on gender.

Variables	Gender	*N*	Mean	Standard deviation	*F*	*P*	ŋ^2^
Collaboration	Male	50	3.53	0.18	0.68	0.41	0.01
	Female	134	3.51	0.21			
Training	Male	50	4.02	0.38	0.68	0.41	0.01
	Female	134	3.97	0.34			
Proficiency	Male	50	2.54	0.18	6.23	0.01	0.03
	Female	134	2.46	0.20			

### 5.3 Mediating effects of teacher collaboration on training and teaching resource proficiency

**Figure 1:**
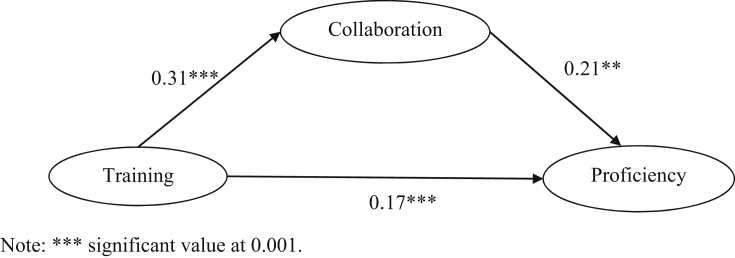
Collaboration as a mediator between teacher training and teaching resource proficiency.

Figure 1 shows the standardised coefficient for the final model. Overall, the model was good (x2/df = 1.18, *p* < 0.001; CFI = 0.99; GFI = 0.99; RMSEA = 0.04). The effect of in-service training on teaching resource proficiency was significant (*β* = 0.17, *p* < 0.001). In-service training had a positive effect on collaboration (*β* = 0.31, *p* < 0.001). Collaboration as a mediator also showed a significant positive impression on teaching resource proficiency (*β* = 0.021, *p* < 0.001). These findings provided evidence that teacher collaboration significantly influences their participation in professional training, which influences improved teaching resource proficiency amongst the Chemistry teachers.

## 6 Discussion

The results show that in-service training amongst Chemistry teachers is at a high level. However, teacher collaboration and teaching resource proficiency are at a moderate level. In general, the Chemistry teachers who participated in the survey have attended relevant professional training, which suits their needs and has assisted them in their tasks. This finding implies that the ministry has provided training that is relevant to the teachers. It also confirms several claims on the significance of self-development through continuous education. Mangkuprawira (2004) described in-service training as a process of providing updated information, skills and attitudes for employees to improve their skills and empower them to perform their responsibilities effectively. From the aspect of collaboration, teachers need help from other teachers to solve their problems and overcome challenges. Sharing of expertise through face-to-face meetings or via technology can guide other teachers to adapt with current progress and trends by focusing on the advancement of knowledge in various aspects. It also encourages teachers to expand their knowledge continuously and find the latest resources. The results also reveal that the Chemistry teachers still need to improve their teaching resource proficiency. Many Chemistry teachers have not yet fully mastered their teaching materials. The effectiveness of teachers' development training activities has a connection to the quality of teaching supervision and assessment (Guskey and Sparks, 1996). Guskey (1990) suggested that any new plan for the development of teachers’ professionalism should focus on strengthening them by evaluating the existing in-service training.

The different proficiency level between male and female teachers is notable. Male teachers are more proficient than female teachers in teaching resources. This situation illustrates that male teachers have mastered their materials, so they have more motivation to conduct laboratory experiments before they start teaching. This statement is in line with Yusrizal et al. (2011), who argued that the outcome of a teacher's work has an association with his motivation. Teachers should have self-motivation to develop their potential and produce satisfactory outcomes in their work. This study proves that male teachers engage more in mastering their Chemistry teaching materials. Thornton and Morony (2005) stated that the standard of teaching comprehensively describes the whole process of improving the teaching profession throughout the service. Hussin (2003) added that educators should equip themselves with the latest knowledge in line with the rapid and various changes in the field of education, including the use of new terminologies and the application of the latest teaching and learning methods. Wise (2004) reported that 91% of teachers with good content knowledge have successfully passed their examinations.

The SEM test results indicate that training on teacher proficiency despite a low percentage of contributions at only 17%. Conclusion can be made that the training organized for the teachers is relevant yet inadequate to enhance teacher proficiency. With regard to the content of the training, it is found that there are several areas which need to be improvised including learning improvement, preparation of teaching aids and materials, innovation in learning activities, ideas for action research and skills in practicum tools. These aspects should be the main concern as there are the major areas of teacher proficiency and skills in teaching and learning, as well as the main task for an educator. Chemistry teachers who have gained extra knowledge and experience from a training would have more opportunities to integrate the knowledge and experience in their teaching and learning. This is due to the fact that they have the ability to relate lesson materials with the current development in accordance with students needs and also in integrating lesson materials in daily life (Lau, 2013). However, supports from the school in terms of providing the facilities needed by the teacher are also important.

Regrettably, the teachers who have received training do not show a significant effect in mastering and integrating such knowledge in their teaching and learning process. Results have found that teacher proficiency is still at a moderate level. This indicates a slight effect of training towards Chemistry teachers. Hence, with regard to the lack of proficiency among the Chemistry teachers, it is paramount to take extra efforts to improve the teachers’ proficiency levels. Among them is to provide better content that is appropriate to the needs of current teachers and provide facilities for the teachers to facilitate the integration of knowledge in the teaching and learning process. In addition, teacher collaboration should also be considered as it is a significant platform for teachers to improve their proficiency (Vangrieken et al., 2015). This revelation is deduced from the results of this study which confirm that teacher collaboration acts as a mediator between training and teacher proficiency. Another conclusion can be made that teacher proficiency can be improved through collaborations among teachers in the same school or with teachers from other schools as well as collaboration with certain organizations.

The SEM test results indicate collaboration is a significant mediator for the relationship between training and teaching resource proficiency amongst Chemistry teachers. The results of this study reveal that teacher collaboration has 21% change effect on the link between training with teacher proficiency. This indicates that training alone is insufficient to improve teacher proficiency. Therefore, the shortcomings of the organized training can be overcome by teacher collaboration. Teacher collaboration enables teachers to share ideas and knowledge, to solve teaching and learning problems, to assist teachers who are incompetent in ICT and to create groups of expert teachers to establish programs that can further enhance teacher proficiency (Mora-Ruano et al., 2018). In fact, perceptions on teacher collaboration are higher than training. This means that teachers are positive towards collaborative efforts as a mean to improve their proficiency. Gudmundsdottir (1990) argued that not all teachers are aware of everything, and some of them follow their interests and wants regarding their subject matters. Nevertheless, collaboration with fellow teachers or referring to experts brings a positive influence on teaching resources proficiency amongst Chemistry teachers.

Some evidence was found on the practice of collaboration in mastering ICT, involvement in programs organized by the school, sharing ideas on the web and explaining work instructions to colleagues. Thus, this study manages to show the existence of collaboration among chemistry teachers. Therefore, further actions must be taken by the school to facilitate and provide a way for teachers to strengthen and widen their collaborative programs with other schools or with relevant organizations. The results provide implications to the Ministry of Education to increase teacher collaboration, in-service training and teaching resource proficiency, especially amongst female teachers. The results show that female teachers have lower collaboration, in-service training and teaching resources proficiency than male teachers. Efforts to emphasize these three aspects, such as providing training that considers the current needs of chemistry teachers, are relevant. The ministry should also consider fostering cooperation and training aspects to improve teacher proficiency. In this context, this study proves that training and collaboration in improving chemistry teacher proficiency. On behalf of teachers, through the results, we expect them to engage in various programmes provided by the ministry and have confidence and motivation to improve their quality as professional teachers.

This study highlights the significance of professional development and networking amongst teachers. Teachers should collaborate and maintain good communication with their colleagues and share their knowledge. This effort can be a stepping stone to enhance teacher proficiency, particularly in mastering their teaching materials. Schools also need to provide adequate facilities for teachers, including the availability of technology, laboratories and chemicals required in Chemistry teaching. This kind of support can ease the formation of a professional learning community. It encourages teachers to discuss with experts freely or explore their abilities in a conducive environment. Authorities should maintain and enhance in-service teacher training because of its direct influence on teaching resource proficiency. They should conduct continuously series of training for teachers to identify their needs in teaching Chemistry subject. Finally, further research should involve various aspects, such as creativity, laboratory skills and ICT skills, to enhance teacher proficiency.

This study contributes to the stakeholders of education including schools and the Ministry of Education to organize better trainings particularly in the areas of learning improvement, preparation of teaching aids and materials, innovation in learning activities, ideas for action research and skills in practicum tools. This study has successfully revealed that teacher collaboration is a significant mediator between training and teacher proficiency. Future studies are recommended to look into matters related to training in the aspects of planning, implementation and its effectiveness. This is pertinent due to the findings that previous trainings only have small contributing effect on teacher proficiency. However, comparisons should be also made according to teaching experience as teachers of all categories are required to improve their proficiency accordingly to be aligned with contemporary needs.

## 7 Conclusion

This study indicates different proficiency levels between male and female teachers, particularly in teaching resource proficiency. However, teachers exhibit no significant difference in terms of collaboration and in-service training. Male and female chemistry teachers should engage in collaborative activities. These activities can help them meet, liaise, communicate and discuss with other professionals to improve their teaching quality and obtain personal proficiency. Moreover, this work identifies the critical role of collaboration, as it provides a platform for teachers to participate in professional training. Collaborative sessions also help teachers enhance their teaching proficiency. Therefore, teachers should know about the significance of professional or academic collaboration. Schools should organise adequate intervention programmes, including professional meetings involving various teachers, educators and teaching experts. In terms of gender differences, male and female teachers should participate equally to overcome gender bias.

## Declarations

### Author contribution statement

Jimmi Copriady: Conceived and designed the experiments, Performed the experiments, Wrote the paper.

Hutkemri Zulnaidi: Analyzed and interpreted the data, Contributed reagents, materials, analysis tools or data; Wrote the paper.

Masnaini Alimin: Performed the experiments; Contributed reagents, materials, analysis tools or data; Wrote the paper.

Sri Wilda Albeta: Performed the experiments; Contributed reagents, materials, analysis tools or data.

### Funding statement

This work was supported by the Faculty of Teacher Training and Education, Riau University.

### Data availability statement

Data will be made available on request.

### Declaration of interests statement

The authors declare no conflict of interest.

### Additional information

No additional information is available for this paper.
